# Carcinomatous Tumor Attached to the Uvula and Posterior Pillar of the Fauces; Removal and Recovery

**Published:** 1852-10

**Authors:** 


### ARTICLE XVIII.

*Carcinomatous Tumor attached to the Uvula and Posterior Pillar of the Fauces; Removal; Recovery.*

Mr. Birkett has lately had under his care, at Guy's Hospital, a case of tumor of the fauces, remarkable for the locality where the disease sprung up, the malignant nature of the affection, the small amount of uneasiness experienced by the patient, and the success which has attended the removal of the growth. The facts are as follows:

A small, active, and intelligent man, fifty-four years of age, was admitted October 1, 1851, in consequence of a tumor growing from the velum pendulum palati. He states that his health has always been good, that he has lived temperately, and that he has followed an occupation connected with the hemp and flax trade. To the dust which arises in turning over these materials, and the irritation it caused in his throat, he attributes the development of the growth to be presently described. Such an origin, however, is problematical.

A few days before admission the patient complained to a surgeon about a sore throat, and upon the latter examining the region, the tumor was for the first time observed; the man, in fact, was perfectly ignorant of its existence; he had, "to be sure," he said, "felt some little difficulty in swallowing, and thought the food did not go down so well as usually, but he had never thought of looking into his throat." No pain in the part had ever been felt.

The tumor hung down into the pharynx, resting upon the back part and root of the tongue, and advancing into the mouth. This is its ordinary position, and it appeared in this situation when the patient first opened his mouth for an examination.

The tumor rested upon the dorsum of the tongue, covering its two posterior thirds; it may be freely moved from side to side, and seems as if it would fall out. When carefully examined, the peduncle by which it is attached appears to be continuous with the right pillars of the fauces on the outer side, and with the right portion of the uvula on the inner. Towards the left border of the tumor, the left half of the uvula could be detected by irritating the mucous membrane, when a slight retraction of a very minute ridge was sufficient evidence of the situation of this organ. The entire surface was covered by mucous membrane, the subjacent vessels of which were enlarged and tortuous, giving rise to the impression that the growth itself might be supplied with large blood-vessels. In the absence of Mr.

Cock, who deputed Mr. Birkett to attend to the case, the latter punctured the swelling, as he thought, from its elastic nature, that it might arise from an obstructed follicle. But Mr. Birkett was disappointed in this supposition, for he obtained sufficient evidence of the internal structure of the tumor to satisfy himself that the new growth was carcinomatous.

A fortnight after admission, Mr. Birkett removed the entire growth by excision, having first passed a ligature through it, so that an assistant might keep it fowards and prevent it, when its peduncle was partially cut through, from falling down upon the epiglottis. Not the slightest hemorrhage of importance ensued, and the trifling loss of blood which occurred was easily arrested with cold water.

The wound was very trivial, extending along the posterior pillar of the fauces chiefly, and in the site of the uvula. It healed in a few days, and the patient left the hospital ten days after the operation.

The man showed himself again four months afterwards, when the pillars of the fauces were found quite perfect; and except the loss of the uvula, in which part there is a slight cicatrix, the region had entirely regained its normal and healthy aspect.

Upon examining the morbid structure there could be no doubt but that it was of a carcinomatous nature; and this opinion, derived from mere ocular inspection, was confirmed by observation assisted by the microscope. As an instrument for assisting diagnosis, the microscope became available, as stated above, in the first examination.

The entire new growth was enveloped by a thin, delicate cyst, seemingly of the sub-mucous cellular tissue. A section exhibited a uniformly smooth, finely mottled, and granular surface; the elements of the tissue were fibres, nucleated bodies, and granular cells. It was of soft consistence, and very easily broken up. A patch of extravasated blood in the centre was, in all probability, caused by transfixing it with the needle and ligature.

M. Lebert, in his admirable and recently published work on cancer, ("Traitè pratique des Maladies Cancereuses," Paris,

1851,) the best work at present on the subject, writes, at page 421:—

"We distinguish two principal forms of cancer of the palate, the most common of which, viz. that of a diffused kind, presents ill-defined limits. . . . . . The second form is more seldom met with, and is of the encysted kind; of this I have seen two examples. The tumor, in such cases, springs up in the submucous tissue, projects above the velum palati, and is even somewhat movable. The size is variable, but I have seen it as large as a pigeon's egg."—*Lancet.*

